# Modified Ultrafiltration During Cardiopulmonary Bypass and Postoperative Course of Pediatric Cardiac Surgery

**DOI:** 10.5812/cardiovascmed.17830

**Published:** 2014-04-01

**Authors:** Mohsen Ziyaeifard, Azin Alizadehasl, Gholamreza Massoumi

**Affiliations:** 1 Rajaie Cardiovascular Medical and Research Center, Iran University of Medical Sciences, Tehran, IR Iran; 2 Echocardiography Research Center, Rajaie Cardiovascular Medical and Research Center, Iran University of Medical Sciences, Tehran, IR Iran; 3 Anesthesiology Department, Isfahan University of Medical Sciences, Isfahan, IR Iran

**Keywords:** Cardiopulmonary Bypass, Pediatric, Thoracic Surgery, Morbidity

## Abstract

**Context:**

The use of cardiopulmonary bypass (CPB) provokes the inflammatory responses associated with ischemic/reperfusion injury, hemodilution and other agents. Exposure of blood cells to the bypass circuit surface starts a systemic inflammatory reaction that may causes post-CPB organ dysfunction, particularly in lungs, heart and brain.

**Evidence Acquisition:**

We investigated in the MEDLINE, PUBMED, and EMBASE databases and Google scholar for every available article in peer reviewed journals between 1987 and 2013, for related subjects to CPB with conventional or modified ultrafiltration (MUF) in pediatrics cardiac surgery patients.

**Results:**

MUF following separation from extracorporeal circulation (ECC) provides well known advantages in children with improvements in the hemodynamic, pulmonary, coagulation and other organs functions. Decrease in blood transfusion, reduction of total body water, and blood loss after surgery, are additional benefits of MUF.

**Conclusions:**

Consequently, MUF has been associated with attenuation of morbidity after pediatric cardiac surgery. In this review, we tried to evaluate the current evidence about MUF on the organ performance and its effect on post-CPB morbidity in pediatric patients.

## 1. Context

Application of cardiopulmonary bypass (CPB) in heart surgery stimulates pulmonary and systemic inflammatory responses (1). It may increase the morbidity and mortality rates after surgery. The use of CPB provokes the inflammatory responses related to ischemic/reperfusion injury, hemodilution and other causes (2). Contact of blood cells with the bypass circuit surface starts a systemic inflammatory reaction that may cause organ dysfunction after CPB, particularly in lungs, heart and brain. The effects of inflammatory response are usually temporary, but these destructive effects potentially trigger complement activation, cytokines, neutrophil stimulation, and endothelial cell activation (3). Inflammation after CPB is especially prominent in lungs (4). The inflammatory reaction markedly augments the permeability of vessel and pulmonary edema, and reduces the cardiopulmonary function. The inflammatory mediators increase in pulmonary secretions, significantly in the post-CPB period, and are correlated with adverse clinical outcomes (4, 5).

A number of measures have been explained in the effort of reducing the inflammatory reaction; include minimal invasive surgery, use of anti-inflammatory drugs, and ultra-filtration throughout surgery (6). Naik et al. explained the last modality, particularly modified ultrafiltration (MUF). This technique was originally applied in the pediatric population (7, 8). MUF is initiated following the completion of extracorporeal circulation (ECC) and provides its well-known advantages in children with improvements in hemodynamic, pulmonary, coagulation and other organs functions. Decrease of blood transfusion requirement as well as reduced total body water and blood loss after the surgery are additional benefits of MUF (9-11). Conversely, in adult patients, the use of MUF is not well investigated, focusing on its effects on pulmonary, coagulation and hemodynamic consequences. Finally, MUF has been associated with attenuation of morbidity in pediatric cardiac surgery (5, 12). In this review, we tried to evaluate the current evidence about MUF on the organ performance and post-CPB morbidity in pediatric patients.

## 2. Evidence Acquisition

Our “search strategy” was investigating in the, PUBMED, and EMBASE databases and Google scholar for every available article in peer reviewed journals between 1987 and 2013, for related subjects to CPB with conventional ultrafiltration or MUF in patients with pediatric cardiac surgery. We also search in keywords related to MUF in congenital heart diseases operations. We assessed case controls, case series, cohorts and clinical trial studies. We focused on the articles totally or partially relevant to CPB with conventional ultrafiltration or MUF in pediatric population. We used the Medical Subject Headings (MeSH) of anesthesia, congenital heart disease, heart-lung or CPB, conventional filtration and pediatric cardiac surgery. Finally, 26 papers were excluded in the critical appraisal process and 65 of the total of 91 collected articles remained for review.

## 3. Results

### 3.1. Modified Ultrafiltration Technique

**Figure 1.**
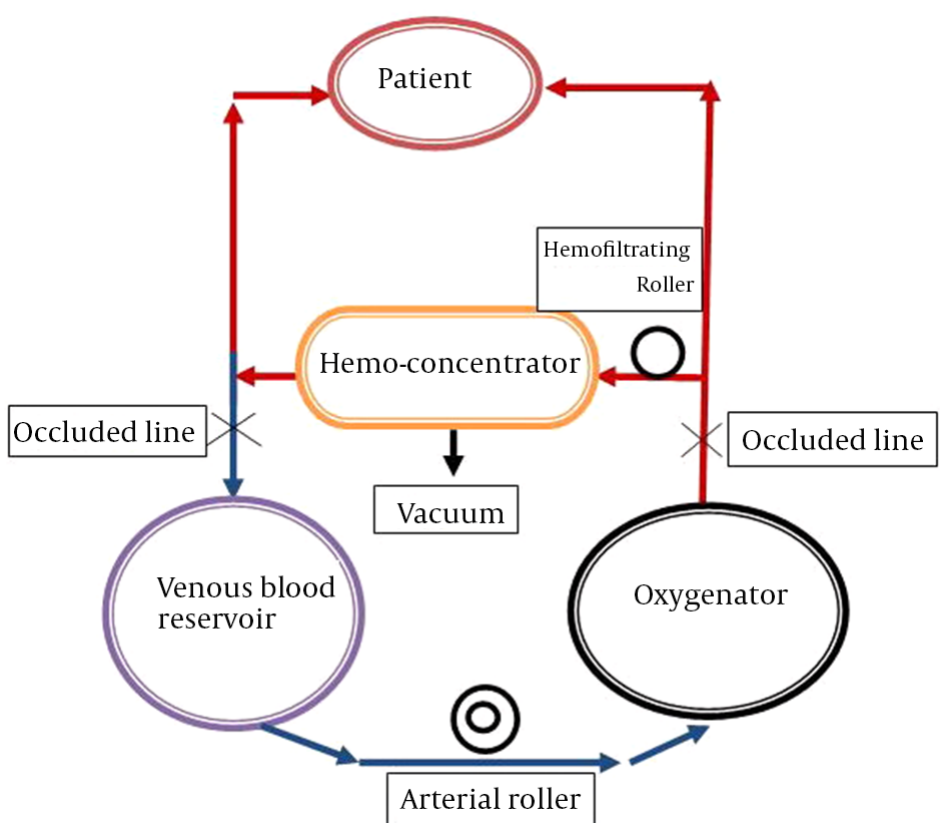
Schematic Illustration of Modified Ultrafiltration

Naik and colleagues applied this technique at the Hospital of Pediatrics in London more than 20 years ago. In this method, the arterial line is connected to the inlet of the ultrafilter and the venous line is connected to the outlet of the ultrafilter in the CPB circuit. The inlet of the filter was clamped throughout the CPB (8). As the patient is separated from the CPB, the clamp is removed from the inlet of the filter, allowing the blood to flow through the arterial line to the filter (10-15 mL/kg/min), and finally from the cardioplegic cannula, as a venous line returns to the right atrium (Figure 1) (13). The filter allows passage of molecules smaller than molecular weight of 65,000 D. When it is needed to maintain the intravascular volume and stabilize the hemodynamics, the blood returns back by the venous reservoir and the venous cannula to the right atrium. This technique was performed until the hematocrit achieved the target of 35% (14).

### 3.2. Inflammatory Response Syndrome and MUF

Acute systemic inflammatory response was induced fallowing surgical trauma and CPB circuit by simulating the cytokine construction, complement activation, coagulation system motivation, activation of neutrophil cells with degranulation, platelet stimulation with aggregation and endothelial cell dysfunction (15-17). Proinflammatory factors, including, interleukin-6 (IL-6), interleukin-1 (IL-1), interleukin-8 (IL-8) and tumor necrosis factor-a (TNF-a), were released during CPB (18, 19). These proinflammatory factors significantly participated in the universal inflammatory reaction syndrome after CPB (5, 20). Several studies have illustrated different consequences. The results of majority of these trials have shown that MUF reduces the amount of circulating inflammatory factors (Table 1).

**Table 1.** The Effects of Modified Ultrafiltration on Systemic Inflammation

Author	Key Results
**Wang et al. (** 21 **)**	Essential reduction in IL-8 and endotelin-1 (ET-1) levels and no alteration in TNF-a level after MUF.
**Bando et al. (** 5 **)**	ET-1 levels were extensively lesser after MUF.
**Pearl et al. (** 22 **)**	MUF does not cause a significant change on thromboxane-B2, ETn-1 and leukotriene-B4 levels post-CPB.
**Portela et al. (** 23 **)**	Essential reduction in levels of IL-6, intercellular adhesion molecule-1and vascular cell adhesion molecule-1 following MUF.
**Yndgaard et al. (** 24 **)**	MUF lower circulatory endotoxins in circulation and recurrence of major amounts of this endotoxin load in the ultrafiltration.
**Chew et al. (** 25 **)**	No intergroup variation visible for TNF-a, IL-1ß and IL-1ra, complements (C3d and C4d).
**Huang et al. (** 19 **)**	Serum IL-6 levels were considerably lower after MUF, mild thromboxane-B2 was removed and ET-1 levels remained unchanged.
**Harig et al. (** 26 **)**	MUF led to lesser platelet activation, monocyte shell markers CD45 and CD14 showed clear generations.
**Yokoyama et al. (** 27 **)**	Removal of prostaglandin E-2 was one cause of augmented blood pressure.
**Atkins et al. (** 12 **)**	MUF was associated with raised alveolar concentrations of proinflammatory cytokines (IL-6 and IL-8) and trans-pulmonary thromboxane meditations.
**Papadopoulos et al. (** 28 **)**	MUF was associated with decrease of inflammatory factors, endotoxins and complements.

### 3.3. Cardiovascular Performance and MUF

The use of CPB in cardiac surgery is a nonphysiologic situation, leading to unfavorable alterations such as hemodynamics changes (27). In the CPB circuit, contact of blood with foreign materials of the tubing surface, activates the leukocytes and leads to release of a range of cytotoxic factors. These factors include proteases, lysosomal hydrolases, arachidonic acid, and some agents that increase the vessels permeability (29). Throughout CPB, controlled hypothermic cardiac arrest may influence the transmembrane fluid transfer due to hypothermia and increase the myocardial interstitial fluid. In addition, ischemia predisposes the heart to pathologic fluid accumulation after return of coronary blood flow (30). Numerous products of cardiovascular activity such as endothelin-1 (ET-1) elevate after CPB (31). Numerous investigations have shown significant improvements of hemodynamic variables after MUF (Table 2) (17, 27, 32-35). These studies have shown improvements in hemodynamic parameters after MUF, including heart rate, systolic and diastolic blood pressure, right and left atrial pressures, pulmonary arterial pressure, and cardiac function. Systemic vascular resistance did not change after MUF, and a remarkable decrease was observed in pulmonary vascular resistance. Hematocrit was increased 25% to 30% after MUF and reduction of ejection fraction was obviously fewer throughout MUF (32-35). Hodges et al. demonstrated that MUF made no change in the depth of anesthesia, because plasma levels of fentanyl remained in the therapeutic range (34). Davies and colleagues showed hemodynamic parameters improvements and decrease in myocardial edema (36). The study of Gaynor and colleagues demonstrated improvement in the left ventricular function that led to reduced need for inotropes in the early postoperative period (37).

**Table 2.** The Effects of MUF on Hemodynamic and Myocardial Functions

Author	Key Results
**Zhou et al. (** 32 **)**	Significant improvement in myocardial function after MUF
**Ricci et al. (** 33 **)**	Significant rise in arterial blood pressure after MUF
**Yokoyama et al. (** 27 **)**	Increased blood pressure after MUF
**Chaturvedi et al. (** 17 **)**	Significant improvement in global left ventricular function after MUF
**Hodges et al. (** 34 **)**	Significant increase in systolic arterial pressure and cardiac index after MUF
**Naik et al. (** 35 **)**	Significant increase in systolic and diastolic blood pressures after MUF

### 3.4. The Effect of MUF on Pulmonary Function

CPB can lead to various degrees of pulmonary injury in most of pediatric patients with severe pulmonary arterial hypertension. Pulmonary dysfunction was marked by reduced pulmonary compliance, increased pulmonary vascular resistance, and also reduced gas exchange (31). Severe acute pulmonary involvement can occasionally lead to fatality (38, 39). CPB hemodilution decreases serum levels of albumin and colloid osmotic force, and augments the efficiency of capillary filtration force. These changes may lead to increase of plasma fluid in the interstitial space, which will reduce the lung compliance and damage the gas exchange through the respiratory membrane. Following aortic cross-clamp, lungs will be ischemic and give off metabolic products to the interstitial liquid. Once the declamping of aorta and the oxygenated blood flow in the lungs creates oxygen free radicals and toxic agent, ischemic-reperfusion injury of the lungs occur. In addition, contact of blood to the CPB circuit as well as hypothermia and hemodynamic fluctuations, promote the discharge of inflammatory factors, leading to an extensive inflammatory reaction causing additional lung injury (38, 40). Several methods have been used to handle the overloaded tissue fluid. Conventional ultrafiltration in CPB (CUF) has been used, which was effective in reducing the water retention after the surgery. Naik et al. descripted that MUF was superior to CUF principally in its capability to decrease the fluid retention related to CPB in pediatrics (35). Numerous trials have shown that MUF may reduce the pulmonary dysfunction in pediatrics populations (Table 3) (18, 19, 41-44).

**Table 3.** The Effects of MUF on Pulmonary Function

Author	Key Results
**Keenan et al. (** 18 **)**	Considerable improvement in dynamic and static lungs compliance immediately after MUF
**Liu et al. (** 41 **)**	Considerable decrease in the mechanical ventilation time and ICU stay and better ventilatory indices in the MUF group
**Onoe et al. (** 42 **)**	MUF may result in better pulmonary function in pediatrics after surgery
**Torina et al. (** 43 **)**	MUF had effects on pulmonary function and transfusion necessities
**Huang et al. (** 19 **)**	Continuous and modified ultrafiltration decreased the lung injury
**Mahmoud et al. (** 44 **)**	MUF improved pulmonary function

On the other hand, results of different studies demonstrated that the use of MUF in the post-CPB period in pediatrics patients can improve the pulmonary compliance and gas exchange, which may successfully reduce the pulmonary dysfunction after the pediatric cardiac surgery. MUF may lead to decreased period of tracheal intubation and mechanical ventilation, ICU stay, and total hospitalization after the surgery (40-44).

### 3.5. The Effect of MUF on Total Body Water

CPB in pediatric cardiac surgery exposes children to severe hypothermia, hemodilution and contact of blood to surface bypass circuit, initiating a systemic inflammatory reaction (45). Total body water (TBW) increases as a result of capillary permeability which often leads to tissue edema and is followed by multiple organ dysfunctions, principally in lungs, heart and brain (8). TBW usually expands 11% to 18% in the post-CPB period (8). Causes of TBW increase include hemodilution, hypothermia, young age and long duration of CPB (46, 47). Some methods have been used to reduce tissue edema and hemodilution after CPB, including ultrafiltration through CPB or conventional ultrafiltration, peritoneal dialysis after the surgery, forceful use of diuretics, and MUF (45, 47). The significantly positive result of MUF is reducing TBW after bypass. Reduction in TBW resulted in the elevation of hematocrit to pre-CPB levels and explained better organ function and reduction in postoperative morbidity (48, 49).

### 3.6. The Effect of MUF on Coagulation and Blood Transfusion Requirements

Post-CPB coagulopathy is a well-known complication (50-52). Some studies described that MUF considerably decreased the CPB-related coagulopathy in children undergoing congenital heart surgery (53-55). Ootaki et al. studied seven infants undergoing pediatric cardiac surgery (54). They explained that MUF was associated with considerable rises in platelet count, hematocrit, total albumin and plasma protein. Furthermore, prothrombin, factor VII and fibrinogen levels elevated considerably by MUF (55, 56). In addition, increase in hematocrit is a reliable effect of MUF. Bleeding and need for transfusion in the postoperative period have multiple factors including hemodilution, fibrinolysis disorder, and platelet activation (11, 56). Several strategies have modified the hemostatic changes after CPB. MUF improved the hemostasis in the post-CPB period with useful effects on the blood loss, chest tube drainage, and blood transfusion requirements after the surgery. Need for blood products including red blood cells, fresh frozen plasma, cryoprecipitates and platelets were considerably lesser in MUF patients compared with the control group of infants (5, 53, 57).

MUF considerably decreased the necessity for blood transfusion in both deep and moderate hypothermia patients (5, 54, 58). MUF has been demonstrated as a reliable and valuable method for haemoconcentration with positive impacts on reducing the blood loss and transfusion requirements after cardiac surgery (8, 59, 60).

### 3.7. Complications of MUF

Complications of MUF were lung air emboli, dysrhythmia, hypothermia, persistent systemic hypotension, and neurological deficits. These were avoided with application of safety strategy for MUF (61, 62). These strategies included continuous arterial line pressure monitoring, use of bypass arterial line filter, application of a bubble trap in the MUF circuit, hemoconcentrator inversion, changing the position of the bubble detector, warming before haemoconcentration, and use of a heat exchanger in the MUF circuit. It was necessary to apply a warm blood for transfusion line in the MUF circuit, regulate the operating room temperature to be adequately warm, consider a positive pressure servo-regulated MUF, apply an ultrasound flow-meter, assign an in-a-row hematocrit sensor, and use a veno-venous ultrafiltration method (63, 64). Additionally, MUF could change the plasma levels of some drugs, including midazolam, fentanyl, alfentanil, aprotinin and heparin (37, 65).

## 4. Conclusions

Use of modified ultrafiltration after separation from CPB can improve the pulmonary compliance and alveolar gas exchange, which may successfully reduce the lung dysfunction after pediatric cardiac surgery. MUF led to decrease of TBW accumulation with improvement in heart and lung function in the post-CPB period in neonates, infants and children. MUF was associated with lower red blood cell transfusion and reduced morbidity. MUF was recognized as a safe and reliable method for haemoconcentration. Use of MUF was not associated with detrimental hemodynamic fluctuation.
